# Bacterial dynamin-like proteins reveal mechanism for membrane fusion

**DOI:** 10.1038/s41467-018-06559-6

**Published:** 2018-09-28

**Authors:** Marc Bramkamp

**Affiliations:** Ludwig-Maximilians-Universität München, Fakultät Biologie, Großhaderner Straße 2-4, 82152 Planegg-Martinsried, Germany

## Abstract

The dynamin superfamily of large GTPases comprises specialized members that catalyze fusion and fission of biological membranes. While fission-specific proteins such as dynamin work as homo-oligomeric complexes, many fusion catalysts such as mitofusins or bacterial dynamin-like proteins (DLPs) act as hetero-oligomers. However, so far it was unclear how these hetero-oligomeric DLPs assemble and how they function in membrane remodeling. The group of Harry Low report now on the structure of a DLP pair from *Campylobacter jejuni*, allowing detailed insight into the assembly mechanism and membrane tethering activity.

## Introduction

Biological membranes are the boundary of all cells and, hence, a prerequisite of cellular life. Integrity and dynamics of membranes are therefore essential and tightly regulated. Naturally, cells with complex membrane systems such as eukaryotic cells have evolved more specialized systems to maintain membrane integrity, compared to structurally more simple cells such as bacteria or archaea.

Fusion and fission of membrane is often regulated by proteins of the dynamin superfamily (DFS)^1–3^. These proteins are large GTPases with additional domains that are required for membrane binding and oligomerization. A hallmark of DSF is the assembly stimulated GTP hydrolysis and G-domain interaction, essential for oligomerization. Structural data suggest that DFS proteins catalyzing membrane fusion and fission can be separated by their domain architecture^1^.

Several DLPs involved in membrane fusion act as hetero-oligomeric complexes. This is the case for mitofusins (MFN), responsible for fusion of the outer membrane of mitochondria, and the isoforms of OPA1, required for mitochondrial inner membrane fusion^4,5^.

## Bacterial DLPs

Most bacterial DLPs are encoded in an operon with two adjacent genes, suggesting that they act as heterotypic complexes similarly to eukaryotic MFN and OPA1. In Firmicute bacteria, a single gene encodes a two-headed DLP. So far it remained mysterious how these proteins act in homotypic membrane tethering and fusion.

Consistent with the idea of catalyzing homotypic membrane tethering and fusion, bacterial DLPs have been associated with several membrane-related functions in vivo such as cytokinesis^6^, membrane vesicle formation^7^ and membrane surveillance and repair^8^. In vitro studies with the *Bacillus subtilis* DLP DynA showed that this protein can induce membrane fusion^9^. Thus, the current model is that bacterial DLPs are recruited to sites were homotypic membrane fusion is required. However, the mode of interaction in heterotypic DLP complexes was unclear and therefore the molecular mechanism how membrane fusion is achieved remained enigmatic.

## A bacterial DLP pair from *Campylobacter jejuni*

**Fig. 1:**
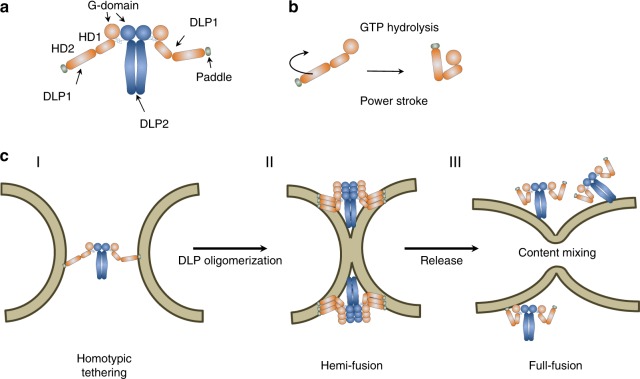
Function of bacterial DLPs in membrane fusion. **a** Cartoon of the *C. jejuni* DLP1/DLP2 hetero-oligomer. DLP2 (blue) forms a central dimer flanked on either site by a subunit of DLP1 (orange). The connection of DLP1 and DLP2 is mediated by a short helix and membrane binding of DLP1 is mediated by a paddle domain at the end of HD2 (olive). **b** Based on structures of BDLP from *N. punctiforme*, domain rotation driven by a GTP-hydrolysis cycle is assumed to be the power stroke for membrane deformation or tethering. **c** Simplified model of bacterial DLP like membrane fusion. (I) A DLP hetero-oligomer tethers homotypic membranes in trans. (II) Conformational rearrangements are the power stroke to bring membrane in close proximity. Thereby, the membrane is likely bend and a hemi-fusion stalk is generated. The rim of this hemi-fusion stalk is occupied by DLP polymers. (III) Release of the DLP may lead to a sudden relaxation of the highly bend membrane and trigger full fusion leading to content mixing

The newly reported structure of a bacterial DLP pair from the pathogenic bacterium *Campylobacter jejuni* allows the first glimpse into the structural organization of oligomeric fusion DSF^10^. The *C*. *jejuni* DLP1/DLP2 pair assembles in a stoichiometry of 2:2, with the DLP2 protein forming a central dimer and DLP1 positioned on either side (Fig. 1a). The overall structure of DLP1 and DLP2 is canonical, with a G-domain separated by a hinge from a neck (HD1) and trunk (HD2) region, forming together a four-helix bundle. The minimal functional unit of DLP1/DLP2 is a tetramer, supporting earlier observations that the *B. subtilis* DynA is dimeric^9^ and thus also consists of four DLP-like subunits. Unlike the criss-cross interaction of stalk regions in dynamin^11^, *C. jejuni* DLP2 forms a large interaction surface along the entire length of the four-helix bundle. Similar to the structure of LeoA, a bacterial DLP encoded in enterotoxigenic *Escherichia coli*^7^, the four-helix bundle of *C. jejuni* DLP2 lacks a distinct hinge region between neck and trunk. The overall structure of DLP1 is comparable to the structure of BDLP from *Nostoc punctiforme*^12,13^. In BDLP and DLP2, the neck (HD1) and trunk (HD2) domains are separated by a hinge region. The trunk region contains the membrane binding domain at its distal tip (Fig. 1a). Structural analysis of various DLPs clearly shows large domain movements around the hinge region. In membrane-bound BDLP the trunk is rotated 135°, resulting in a stretched conformation. In the apo conformation this hinge is fully closed^13^. Partial structures of MFN1 reveal the existence of a four-helix bundle similar to that of bacterial DLPs linked to the G-domain^14,15^. Nucleotide binding to MFN1 also induces a 77 °C movement of the HD1 domain with respect to the G-domain^14^, indicating a conserved conformational switch in membrane fusion DLPs. These large domain movements were not observed in structures of dynamin, but a recent study also from the lab of Harry Low reports a full length apo structure of the mitochondrial fission DLP Dnm1, with a closed hinge 1 leading to a compaction of the G-domain against the stalk, suggesting that hinge 1 closure could be a conserved feature in in DSF proteins^16^. The flick knife closure of fusion DLPs likely constitutes the powers stroke that brings opposing membranes in close proximity or allow membrane bending to promote fission (Fig. 1b).

Importantly, the structure of the *C. jejuni* DLP1/DLP2 hetero complex reveals how the tight interactions between the two proteins is achieved. A short, 16 amino acid long N-terminal helix on DLP2 forms a connection to DLP1, where this helix is inserted into a groove within the DLP1 assembly domain (a globular domain located between G-domain and neck). It seems plausible that this connection may be able to transfer information on nucleotide state and conformation between DLP1 and DLP2^10^. This linker allows G-domain dimer formation and assembly-stimulated GTP hydrolysis, a hallmark of DSF proteins^2^.

## Outstanding questions

The cellular function of *C. jejuni* DLP1/DLP2 is currently not known, but the solved structure is in agreement with a function in membrane tethering and fusion. The interesting question is: why are many DLPs acting as hetero-oligomers? Although, we still lack data to fully answer this question, there is good evidence for a division of labor. *C. jejuni* DLP1 and the D1 domains of DynA mediate membrane binding, while *C. jejuni* DLP2 and the D2 domains of DynA do not (or only weakly) bind to membrane. Thus, it is plausible that the second DLP may rather serve a regulatory or stabilizing role. Upon polymerization of the DLP complexes along the membrane, the DLP2 part could stabilize the membrane association, or regulate the nucleotide hydrolysis cycle. Indeed, GTP hydrolysis requires two active GTPase sites as shown for DynA and *C. jejuni* DLP1/DLP2^9,10^.

Based on the new structural data and the existing biochemical evidence, a model of how the heterotypic DLP1/DLP2 pair works in membrane tethering can be formulated (Fig. 1c). DLP1 adopts an extended conformation, allowing reaching out for membranes that are distantly spaced. Since the DLP1 protein binds with high affinity to membranes, the DLP complex allows for efficient membrane tethering. Based on the structures of BLDP and MFN1 in their holo- and apo-conformations, it seems plausible that large conformational rearrangements occur upon GTP hydrolysis, leading to a closing of the extended conformation of the four-helix bundle^10,13,14^. This conformational change would provide the power stroke for bringing membranes in proximity. Additionally, polymerization of DLPs at membrane contact sites could lead to membrane crowding and subsequent bending. Extreme bending brings membranes in a highly fusogenic state, and the outer leaflets of the opposing membranes can merge, forming a hemi-fusion stalk (Fig. 1c). It is likely that the DLPs assemble at the rim of this hemi-fusion ring. It is still speculative how the fusion of the inner leaflet is achieved, but evidence accumulates that the rapid release of the DLP collar at the fusion rim leads to a quick relaxation of the membrane and to full fusion (Fig. 1c).

It becomes clear that, in many cases of membrane fusion, two DLPs are needed to act as hetero-oligomer to catalyze membrane fusion. The new work form Low and colleagues has provided us with the first structural view on how these oligomers may be formed and how this leads to efficient membrane tethering. This new insight also raises important questions. We still do not know the exact mechanism of how DLPs achieve membrane fusion at a molecular level, but the new structural data of the full length DLP1/DLP2 heterotypic oligomer will allow designing experiments that address this question in a detail that were not possible before. Protein dynamic studies, maybe using inter- and intramolecular FRET will help to get insight into the structural rearrangements that occur during the tethering, fusion and release process of DLPs. In particular, the molecular dynamics of the DLP2 part remains to be analyzed. Studies on bacterial DLPs are getting us closer to understand fusion processes and may help to explain fusion processes in general, including fusion of eukaryotic organelles and vesicles. Thus, after all, research on bacterial model systems takes a driver seat in cellular biochemistry and cell biology.
